# Hemp-Based Microfluidics

**DOI:** 10.3390/mi12020182

**Published:** 2021-02-12

**Authors:** Mikail Temirel, Sajjad Rahmani Dabbagh, Savas Tasoglu

**Affiliations:** 1 Department of Biomedical Engineering, University of Connecticut, Storrs, CT 06269, USA; mikail.temirel@uconn.edu; 2 Department of Mechanical Engineering, Koç University, Sariyer, Istanbul 34450, Turkey; sdabbagh19@ku.edu.tr; 3 Koç University Arçelik Research Center for Creative Industries (KUAR), Koç University, Sariyer, Istanbul 34450, Turkey; 4 Boğaziçi Institute of Biomedical Engineering, Boğaziçi University, Çengelköy, Istanbul 34684, Turkey; 5 Koc University Research Center for Translational Medicine, Koç University, Sariyer, Istanbul 34450, Turkey; 6 Center for Life Sciences and Technologies, Bogazici University, Bebek, Istanbul 34470, Turkey

**Keywords:** hemp, microfluidics, paper, diagnostics, urine diagnostics

## Abstract

Hemp is a sustainable, recyclable, and high-yield annual crop that can be used to produce textiles, plastics, composites, concrete, fibers, biofuels, bionutrients, and paper. The integration of microfluidic paper-based analytical devices (µPADs) with hemp paper can improve the environmental friendliness and high-throughputness of µPADs. However, there is a lack of sufficient scientific studies exploring the functionality, pros, and cons of hemp as a substrate for µPADs. Herein, we used a desktop pen plotter and commercial markers to pattern hydrophobic barriers on hemp paper, in a single step, in order to characterize the ability of markers to form water-resistant patterns on hemp. In addition, since a higher resolution results in densely packed, cost-effective devices with a minimized need for costly reagents, we examined the smallest and thinnest water-resistant patterns plottable on hemp-based papers. Furthermore, the wicking speed and distance of fluids with different viscosities on Whatman No. 1 and hemp papers were compared. Additionally, the wettability of hemp and Whatman grade 1 paper was compared by measuring their contact angles. Besides, the effects of various channel sizes, as well as the number of branches, on the wicking distance of the channeled hemp paper was studied. The governing equations for the wicking distance on channels with laser-cut and hydrophobic side boundaries are presented and were evaluated with our experimental data, elucidating the applicability of the modified Washburn equation for modeling the wicking distance of fluids on hemp paper-based microfluidic devices. Finally, we validated hemp paper as a substrate for the detection and analysis of the potassium concentration in artificial urine.

## 1. Introduction

Global warming [1], climate change [2], the escalating demand for energy [3], the depletion of crude oil resources [4], pollution [5], deforestation [6], and growing concerns regarding environmental crises [7] have drawn the attention of both industry and academia to altering nonrenewable resources with biobased materials with low environmental impact [8,9,10,11,12,13,14]. Hemp (*Cannabis sativa*) is an annual plant that has applications in the food and beverage industry [15], cosmetic products [15], the textile industry [16], thermal insulators [17,18], garden products [19], biofuels [20], bio-oils [21], biofibers [22], paper fabrication [23], the refinement of water and sewage [24], nutritional supplements [25], reinforcing composites and plastics [26,27], and phytoremediation [15,28] (i.e., removing heavy metals from contaminated soil by cultivating plants). Hemp is one of the oldest plants cultivated by humans for its oil, seeds, and fiber [29], dating back 12,000 years in China, probably introduced to Europe during the Iron Age [30]. Interestingly, the Guttenberg Bible (1456) [15] and the first draft of the Declaration of Independence (1776) were printed onto hemp papers [15,31]. However, the emergence of synthetic resins and composites as well as the ease of producing paper from forests diminished the demand for hemp [30]. Finally, the cultivation of hemp was banned in the 20th century due to the plausible usage as an opiate drug [15,30].

**Figure 1:**
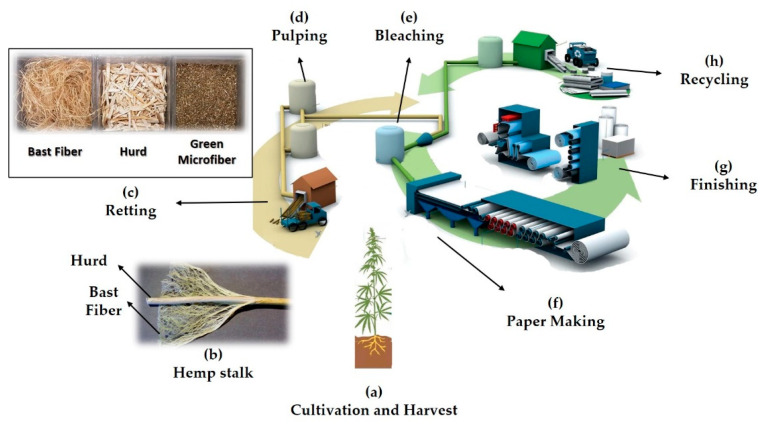
Hemp paper lifecycle. (**a**) The hemp plant can be cultivated on either side of the equator, preferably between 25th and 55th latitude parallels [31]. Seeds, leaves, and stalk are the three main parts of the hemp plant. (**b**) After cultivation, the hemp stalk should be split apart from roots and leaves. The stalk is comprised of two parts: woody core (hurd) and bast fiber. Bast fiber possesses 57–77% cellulose content and tougher fibers compared to hurd, with lower cellulose content (40–48%) [31,33]. (**c**) The retting process breaks the chemical bonds that keep hurd and bast fiber together [34,35]. The separated bast fiber, hurd, and green microfibers have different applications [15]. Bast fiber can be used to fabricate ropes, textiles, cloths [16], concrete, composites [18], and reinforced plastics [36,37,38,39]. Hurd has applications in the production of paper [18], fuel [20], hempcrete [40,41], fiberboard [42,43], insulations [17,18], pet litter [19], garden products [19], and food preservation. Moreover, bionutrients, beauty products, and pet wellness feedstock can be derived from hemp seeds and green microfiber [15]. (**d**) Besides cellulose, lignin is one of the most important constituents of hemp. Pulping is the process of degrading semicellulose and lignin to small, water-soluble molecules that can be washed out [44]. Although chemical pulping removes lignin and other impurities more efficiently, the use of chemicals raises concerns regarding environmental issues [31,44]. Mechanical pulping, on the other hand, decreases the use of chemicals, but the quality of the resultant pulp is not as good as with chemical pulping [45]. Biopulping is an emerging technique that can substantially address the drawbacks of both chemical and mechanical pulping [44]. Chemical pulping can be carried out by kraft, soda or soda anthraquinone, neutral sulfite, and acidic sulfite processes [31]. Stone groundwood, refiner mechanical (RMP), thermomechanical (TMP), and chemimechanical (CTMP) are the most common mechanical pulping methods [45]. (**e**) Even after the pulping process, the pulp contains remnants of lignin and diverse chromophoric compounds [31]. In order to produce high-quality, brighter, whiter, softer, and highly absorbent papers, a bleaching step is necessary to remove the remaining lignin [45]. This process can be performed using different chemicals, namely, chlorine, sodium hypochlorite, caustic soda, chlorine dioxide, oxygen, ozone, hydrogen peroxide, peracetic acid, and enzymes [31]. Unbleached pulps can be used in the production of linerboard, boxboard, and grocery bags. Bleaching of mechanical pulps results in lower-grade papers, turning to yellowish colors over time, suitable for newspapers and pocket fabrication. However, bleaching of chemical pulps does not induce a yellowish paper problem [45]. (**f**) The paper-making step includes screening, cleaning, and deforming the bleached pulp into paper rolls using pressing rollers. Screening and cleaning are performed to remove unwanted oversized particles (debris) from paper pulp. Debris refers to shives (small fiber bundles that have not been separated by mechanical or chemical pulping), chop (oversized particles), and knots (uncooked particles) [45]. (**g**) The finishing step is performed to prepare papers with the desired size and design. (**h**) Hemp paper can be recycled up to 8 times, compared to 3 times for wooden paper. Firstly, used papers are rewetted and returned to pulp form. Then, physical objects (e.g., staples) are separated by a mechanical process (e.g., magnets). The next step is deinking papers using chemicals to produce cleaner pulp. The deinking step can be omitted from the recycling process in the case that low-quality paper is needed (e.g., carton and corrugated board production). Finally, the repulped paper can be mixed with virgin pulp to be bleached to reenter the paper-making step [45].

Recently, however, numerous advantages of hemp over wood and synthetic materials have attracted more attention to this plant once again, partially due to its features including sustainability, recyclability, and high yield capacity. Hemp grows to its full size in 3–4 months, whereas it takes 20–80 years for trees [32]. Additionally, hemp yields 3–4 times more fiber than trees per hectare [32], with almost no need for pesticides, preventing soil and underground water contamination [33]. With more resistance to droughts, hemp contains a higher level of cellulose compared to wood, pointing out the superiority of hemp for paper production [33]. Furthermore, hemp papers can be recycled up to eight times, compared to only three times for wooden papers [32]. Hemp papers have more resistance to decomposition and becoming yellowish in the course of time [32] (Figure 1).

The main drawbacks of hemp-based fibers and composites, or biocomposites in general, are poor adhesion to synthetic polymers [46], degradation during curing at high temperatures [9], and humidity absorption, resulting in weaker mechanical properties compared to synthetic materials [37]. However, several studies have been conducted successfully to address the drawbacks of biocomposites by chemical as well as physical treatments and surface modifications [9,30]. Commonly used physical methods are clantering, stretching, thermotreatment, electric discharge, the production of hybrid yarns, cold plasma treatment, corona treatment, and alkalization [30]. The chemical methods include esterification, liquid ammonia, the silane coupling method, permengnates, isocynates, and graft copolymerization [30]. Furthermore, although integrating biocomposites with synthetic materials reduces the biodegradability of the final product, this hybridization brought about flexibility in design, better mechanical properties, higher impact strength [9,47], better thermal isolation [48], the capability of sound abatement [49], and lower density [46]. Overall, considering the advantages and drawbacks, hemp is a promising choice for biofuel production, oil extraction, the fabrication of interiors of automobiles, and biomedical applications, such as paper-based and polymeric microfluidic devices [19].

Microfluidic analytical devices have a wide range of applications in point-of-care diagnosis [50,51,52,53,54,55,56], clinical tests [57,58,59,60,61], food safety measurements [62], and environmental protection [63,64]. Although conventional laboratory-based analytical devices can be employed for these applications, microfluidic devices are designed to be portable and affordable, and can perform multiple tests on one device [65,66]. Laser etching [67], photolithography [68,69], plasma treatment [70], and 3D printing [71,72,73,74,75] are widely used to fabricate polymeric, glass-based, and silicone-based microfluidic devices [76,77]. However, these fabrication techniques require long design and implementation procedures and trained experts as well as expensive equipment and chemicals, challenging their suitability for mass production [76]. Using paper, on the other hand, as the base material for microfluidic devices [78,79], not only can offer the common advantages of conventional microfluidic devices, but also adds cost-effectiveness, ease of use and fabrication, rapid prototyping, biocompatibility, disposability, and no need for external power sources (e.g., pumps) [76,80]. Therefore, integrating microfluidic paper-based analytical devices (μPADs) with hemp biofibers can bring numerous advantages for producing affordable, sustainable, environmentally friendly, and functional devices for clinical as well as point-of-care applications.

Here, we propose and studied hemp paper as a substrate for the field of paper-based microfluidics. Although capillary-based microfluidic devices (apart from ordinary paper) have been reported in the literature (cotton-, cellulose-, woven-, and polymer-fiber-based) [81,82,83,84,85], to the best of our knowledge, there is no report of the use and validation of hemp paper for microfluidic applications. In this study, we investigated the water-resistant capability of several markers and characterized the effects of plotting speed as well as pattern dimensions using the pen-plotter-based approach [86], which provides single-step high-throughput patterning of hydrophobic barriers across a hemp substrate. We also characterized the wettability of hemp paper by measuring its contact angle and comparing it to that of Whatman grade 1 (WG1) paper. Moreover, we characterized the dynamics of fluid movement through hemp paper via quantifying the effects of the width and number of branches of the channel on the fluid flow through the channel as well as the effect of fluid viscosity. Additionally, we showed the impact of a hydrophobic side boundary on the imbibition of fluid into the microfluidic device. A mathematical model [87] was tested to predict the imbibition properties by comparing the experimental results. Finally, we validated hemp paper as a substrate for the detection and analysis of the potassium concentration in artificial urine.

## 2. Materials

A desktop pen plotter, AxiDraw (Evil Mad Scientist Laboratories, CA, USA); a laser cutter (VLS2.30 CO2 laser cutter; Universal Laser Systems, Inc., Scottsdale, AZ, USA); Deli markers with different colors (Deli group, Zhejiang, China); double-ended Comix markers (Comix group Co. Ltd., Shenzhen, China), where the fine-tip and broad-tip markers are 0.5 and 2.0 mm in diameter, respectively; 100% hemp handmade paper (Hemp Traders, Paramount, CA, USA); chromatography paper, Whatman No. 1 (GE healthcare life sciences, IL, USA).

## 3. Methods

**The effects of markers, the plotting speed, and the pattern dimensions.** To investigate the capability of the markers to create hydrophobic barriers resisting aqueous solutions on the hemp paper, we tested two commercial brands of markers including Deli and Comix (permanent ink). The patterns, circles with a diameter of 4 mm, were drawn with SolidWorks and plotted with the AxiDraw pen plotter on hemp paper. The water-resistant performance of the patterns plotted with two different colors (red and black) of Deli markers, and one color of double-ended Comix markers (both fine and broad tips) were investigated by spotting an aqueous solution of red food dye in the centers of the patterns. To increase the amount of ink diffusing through the paper, multiple passes of plotting were performed.

AxiDraw is a desktop pen plotter with an XY resolution of 80 steps per mm, a ±0.1 mm reproducibility, and a maximum plotting speed of 177 mm/s [88]. The speed is adjustable, directly affecting the amount of ink diffused through the paper and the feature size of the plotted patterns. Multiple speeds of AxiDraw, ranging from minimum (1%) to maximum (110%), were tested for plotting circle patterns (4 mm in diameter) with 1 and 3 passes to determine the best speed and number of passes. In addition, the thinnest boundary thickness and the smallest diameter circular pattern were characterized by plotting the pattern with different plotting speeds and multiple circle diameters with a Comix-fine tip, respectively. 

**Fluid dynamic characterization.** In order to characterize the fluid flow through the paper channel, we performed fluid dynamic characterization for hemp paper as well as WG1 paper to show the effects of the channel width, the number of branches, and the fluid viscosity on the wicking distance over time. Channel patterns on the paper were designed using SolidWorks and laser-cut with the laser cutter (power, 30%; speed, 30%; *z*-axis, 0.07”). The paper channel widths were varied from 1 to 5 mm, with changing the channel length from 20 to 35 mm, while the number of branches was varied from 1 to 4, with a constant channel width of 2 mm. We prepared solutions with various viscosities, ranging from 1.07 to 8.75 mPa.s, by mixing D-(+)-glucose and ID water in accordance with [89]. Then, the paper channels were vertically held using a clamp on a leveled surface. Subsequently, 200 µL of the solution was applied to the bottom of the paper channel. A video was recorded with a digital camera until the fluid flow nearly stopped in the channel. Thenceforth, image processing was performed via ImageJ to measure the wicking distance over time.

**Imbibition test.** We used the hemp paper and WG1 paper for the imbibition test to identify the effect of a hydrophobic side boundary on the imbibition of the paper. As previously described, paper channels were prepared by either laser cutting or plotting the hydrophobic barriers with Comix ink for both papers. Then, the paper channels were placed in an upright position using a clamp, and 200 µL of DI water was put on the bottom of the paper over the test bench. Right before immersing the paper into water, we started recording a video to measure the imbibition length for 300 s. Image processing was performed via ImageJ.

**Artificial urine.** We prepared an artificial urine solution following our earlier work [90]. To measure the concentration of K+ cations, we used potassium-binding benzofuran isophthalate (PBFI) potassium-sensitive fluorescent probes. Hemp paper was utilized as the reaction medium on which we added the PBFI potassium-sensitive dye solutions with concentrations of 25 μM. We diluted PBFI in DMSO, as it prevents hydrolysis. We employed a microplate reader to quantify the fluorescence intensities of the PBFI potassium-sensitive fluorescent probes. We cut the hemp paper into circular shapes (Ø = 0.25 in) via a CO_2_ laser cutter (VersaLaser, Scottsdale, AZ). We deposited 2 μL of PBFI on the hemp matrix, followed by 2 μL of the metal ion solution. The hemp matrices were then positioned inside the 96-well plates. The excitation/emission peaks were 360/450 nm.

## 4. Results and Discussion

**Figure 2:**
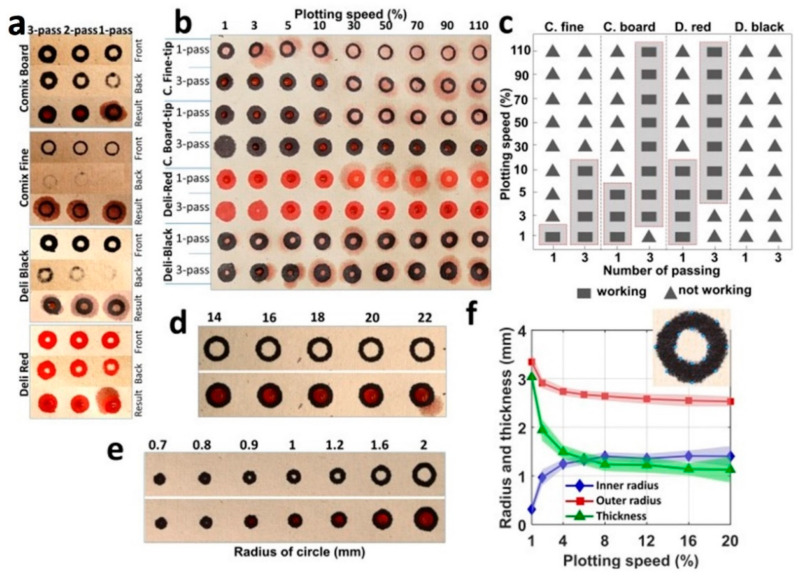
The water-resistant capability of markers and characterization of plotting speed and pattern dimensions. (**a**) Images of 4 mm-in-diameter patterns with Comix and Deli brand markers. From top to bottom, images are arranged to show the front and the back of the hemp papers (only with inks). In order to achieve water-resistant patterns, ink should penetrate to the backside of the paper, preventing the diffusion of applied samples. The bottom rows show results from spotting red aqueous food dye. (**b**) Characterization of the influence of different plotting speeds (left to right), markers, and numbers of passes on the water-resistant capacity of hemp paper. A red aqueous solution was applied to test the plotted patterns. (**c**) Performance chart for different plotting speeds, markers, and numbers of passes; summary of part b. (**d**) Images of patterns plotted with Comix fine tip, with 2 passes at various plotting speeds, to find the thinnest water-resistant boundary thickness. The top image is plotted patterns, and the bottom image is the same patterns after spotting dye. (**e**) Testing multiple circle diameters with Comix fine tip at speed of 20% and 2 passes to find the smallest achievable pattern, without filling by ink. The top image is plotted patterns, and the bottom image is the same patterns after spotting dye. (**f**) Inset: representative image for measuring thickness, and the inner and outer radii of the plotted circles at eight different points (at angles of 0–315° with an interval of 45°) with MATLAB. The mean and standard deviation of inner and outer radii of the patterns plotted at different plotting speeds with 2 passes using the Comix fine tip (n = 3).

**The water-resistant capability of the markers and the characterization of the plotting speed and pattern dimensions.** In this study, we plotted 4 mm-in-diameter circle patterns on the hemp paper with the maximum speed of the plotter (110%), with Comix and Deli brand permanent markers (Figure 2a). A number of the patterns plotted on the front of the paper with the Comix-fine tip and Deli-black were not clearly visible from the back, showing that an inadequate amount of ink penetrated through the paper. According to the bottom row of images in Figure 2a, the diffusion of red aqueous food dye out of the patterned circles elucidates that these two markers were not water-resistant in all the test cases with three passes at maximum speed. The results demonstrate that only the patterns plotted with the Comix-broad tip and Deli-red markers for two and three passes, at a 110% plotter speed, were able to resist the aqueous solution on the hemp paper (Figure 2a).

The effects of the plotting speed and number of passes, for 4 mm-in-diameter circular patterns plotted with different markers on the hemp paper, are illustrated in Figure 2b and Figure S1. The amount of diffused ink and, thereby, the likelihood of achieving water-resistant patterns can be augmented by decreasing the printing speed and/or increasing the number of passes. Figure 2b shows that plotting on hemp paper using the Comix-fine tip marker at a speed of 1% yields a water-resistant pattern. Additionally, by increasing the number of passes to three, water-resistant patterns can be achieved at a speed as high as 10%, which is similar to the results for the Comix-board tip for one pass. However, using the Comix-broad tip and Deli-red markers for plotting can produce water-resistant patterns at all speeds for three passes, except for the speed of 1%, which resulted in ink-filled patterns. Plotting with Deli-black, on the other hand, did not produce a water-resistant pattern for all speeds of multiple passes. Overall, the Comix-board tip and Deli-black showed the best water-resistance performance. Figure 2c summarizes the performance of the markers in creating hydrophobic barriers on the hemp paper.

Even though the Comix-board tip and Deli-red showed the best performance, the thickness of the hydrophobic boundaries was disproportionately thick, even for one pass, in terms of achieving higher resolutions. To find the thinnest boundary thickness, the Comix-fine tip with two passes was tested for different plotting speeds, ranging from 14 to 22%, by plotting 4 mm-in-diameter circle patterns (Figure 2d). We obtained the thinnest water-resistant boundary at a speed of 20% for the Comix-fine tip with two passes. In addition to optimizing the number of passes and plotting speed, to obtain the thinnest water-resistant boundary, we tested multiple circle diameters, varying from 0.7 to 2 mm, to determine the highest achievable resolution, with AxiDraw, for producing water-resistant patterns that are not ink-filled (Figure 2e). According to our results, the smallest feature that can be plotted, without being filled with ink, is a circle with a radius of 1 mm at the speed of 20% with two passes. Figure 2f illustrates the effects of the plotting speeds on the inner radius, outer radius, and line thickness of the plotted patterns, with a diameter of 4 mm, using the Comix-fine tip at a speed of 20% and two passes. Based on this figure, an increase in plotting speed decreased the outer radius and thickness, whereas the inner radius increased. We analyzed the captured images after plotting, with a MATLAB script, to measure the inner and outer radii of the plotted circles and the thickness (i.e., the differences between the outer radius and inner radius) at eight different points (shown as an inset image (at angles of 0–315° with an interval of 45°)) (Figure 2f).

**Figure 3:**
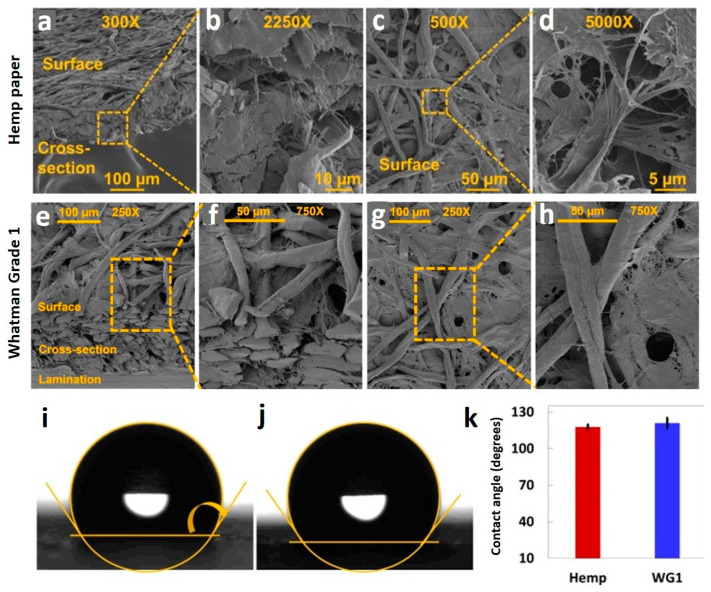
Physical characterization of hemp paper and Whatman grade 1 (WG1) paper. (**a**) SEM image with 300× magnification, showing the surface and cross-section of hemp paper. (**b**) The cross-section of hemp paper with 2250× magnification. SEM images of the surface of hemp paper with (**c**) 500× magnification and (**d**) 5000× magnification. (**e**) SEM image of surface and cross-section of WG1 paper with 250× magnification and (**f**) 750× magnification. (**g**) SEM image of the surface of WG1 paper with 250× magnification and (**h**) 750× magnification [88]. Images of a water droplet (**i**) on hemp paper, and (**j**) WG1 paper; substrates were coated by Comix ink. (**k**) Quantitative results for contact angles for hemp paper and WG1 paper. The contact angle of water on hemp paper is 4% less than that on WG1 paper, demonstrating comparable hydrophobic features of hemp paper and WG1 paper. Error bars represent standard deviation (n = 3).

**Physical characterization.**Figure 3a,b display scanning electron microscope (SEM) images of the isometric view of the surface and cross-section of the hemp paper at 300× magnification, and focused cross-section of the hemp paper at 2250× magnification, respectively. In order to characterize the pore structure and cellulose fibers, the surface of the hemp paper was also imaged at 500× and 5000× magnification, as presented in Figure 3c,d, respectively. Figure 3e–h show SEM images surface and cross-section of WG1 paper under different magnifications. In addition, we measured the contact angle of water on both the hemp paper and WG1 paper by coating the paper surface with Comix ink, as illustrated in Figure 3i,j, respectively. The quantitative results are shown in Figure 3k. The contact angle of water on the hemp paper was about 4% less than that on the WG1 paper. The result proves that hemp paper has a significant capacity, as capable as WG1 paper, regarding the hydrophobic feature of the paper.

**Figure 4:**
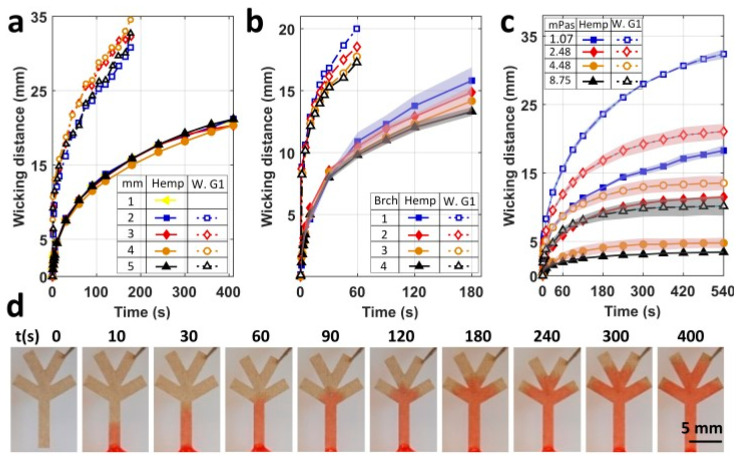
Fluid dynamic characterization of hemp paper and Whatman grade 1 (WG1) paper at room temperature. (**a**) Wicking distances for hemp paper and WG1 paper for various channel widths from 1.0 to 5.0 mm (WG1′s result is from the article [91]). (**b**) Wicking distances in a channel with multiple numbers of branches, from 1 to 4, within 60 s for WG1 (in accordance with [91]) and 180 s for hemp paper. The width of the channel in each branch was 2 mm, and 20 μL sample volume was applied. (**c**) The effect of fluid viscosity on wicking distance in hemp and WG1 strips (2 mm in width). Fluid viscosities were arranged according to [89] by changing the amount of d-glucose in DI water. (**d**) Images of a four-channel, 2 mm-in-width hemp paper strip, where a red aqueous solution was used to show fluid diffusion as a function of time. Error bars represent standard deviation (n = 3).

**The results of the fluid dynamic characterization.**Figure 4 depicts the fluid dynamic characterization of the hemp paper and WG1 paper at room temperature. Figure 4a shows the effect of the channel width on the wicking distance, over 400 s, for the hemp paper. In addition, it shows a comparison of our experimental results for hemp paper with experimental results for WG1 paper from [91], in terms of the impact of the channel width on the wicking distance. As seen in Figure 4a, similar to for WG1 paper, the channel width has no significant influence on the wicking distance on hemp paper, so the wicking distance was virtually equal for channel widths varying from 1 to 5 mm. The underlying reason is that a unit square piece of paper, in the vertical direction, absorbs and transfers the liquid to the upper unit piece in equal time compared to its neighbors in the horizontal direction. Thus, the wicking speed in the vertical direction is independent of the channel width. Additionally, it was observed that WG1 paper absorbs water faster than hemp paper, potentially due to its larger pore structure as well as higher absorbance capacity. Figure S2 shows representative images of fluid wicking on hemp paper with varying channel widths for 410 s. For further fluid dynamic characterizations, channels with a width of 2 mm were chosen. 

The effect of branching channels on the fluid flow through the paper channel was also characterized. Figure 4b illustrates the wicking distances of multiple numbers of branches, from 1 to 4, for 180 s for hemp paper, and for 60 s for WG1 [91], with a 2 mm constant channel width. The WG1 paper had a faster diffusion speed than the hemp paper because of its higher absorbing capacity. Increasing the number of branches from 1 to 4 led to no significant differences in the wicking distance for the first 60 s on the hemp paper. However, after 180 s, the wicking distance for a single channel was approximately 18% more than a four-branch channel. Likewise, the WG1 paper experienced a 14% decrease in the wicking distance as a result of branching, demonstrating performance of hemp paper similar to that of WG1 paper, a commonly used substrate for paper-based microfluidic devices. Figure S3 illustrates images of the fluid wicking distance on the hemp paper, over 400 s, with the number of branches varying from 1 to 4.

Since paper-based microfluidic devices are used for different fluids with various viscosities, the viscosity of the solution is a decisive factor for the efficiency of paper-based devices. In order to characterize the effect of fluid viscosity on the wicking distance, either for hemp paper or WG1 paper, we tested varying viscosities ranging from 1.07 to 8.75 mPa.s (Figure 4c). The wicking distance decreased with increasing fluid viscosity for both papers. The wicking distance of the highest-viscosity solution (8.75 mPa.s) compared to that of the lowest-viscosity solution (1.07 mPa.s) was 83.3% lower on hemp paper and 68% lower for WG1 paper. For the solution with the lowest viscosity, 5 min was needed to reach a 15 mm wicking distance on the hemp paper, explaining the potency of hemp paper for the analysis of low-viscosity solutions such as urine. Figure S4 shows representative photographs of the wicking of solutions with different viscosities in the microfluidic channel made from hemp paper. Figure 4d displays images of fluid wicking through a hemp paper-based microfluidic channel with four branches. The red dye was mixed with the solution for better visualization.

**Figure 5:**
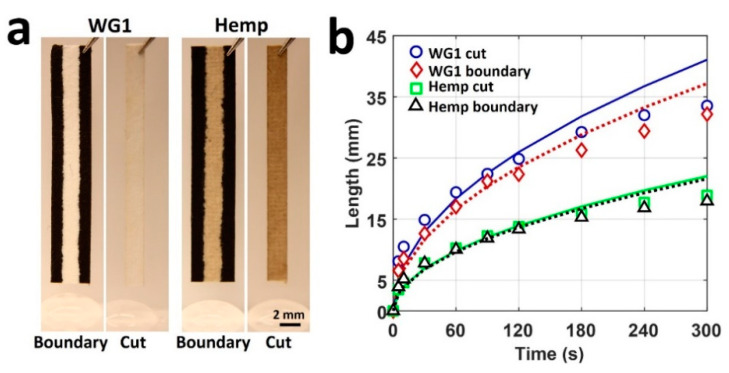
A comparison of experimental results and mathematical model. (**a**) Representative images of hydrophobic boundary and cut boundary channels for both WG1 and hemp paper. The channel widths were 2 mm. (**b**) Comparison of experimental results for wicking length with the mathematical model using Equations (1) and (2). Markers show the experimental data over 5 min. The dotted and solid lines represent the best-fit curve for estimating the constants k and β, respectively, for both papers. A 2 mm paper channel width was used in all experiments (n = 3).

Although we showed that the wicking distance is independent of the channel width for paper channels with laser-cut boundaries, the channel width affects the wicking distance for paper channels with hydrophobic side boundaries. Narrowing down the width of a channel with hydrophobic side boundaries results in decreasing the wicking speed and distance [87]. While the Washburn Equation (1) is valid for laser-cut paper channels, in order to apply the Washburn equation to narrow paper-based channels with hydrophobic side boundaries, this equation needs to be modified (2) [87]. The Washburn equation and modified Washburn equation are as follows:

(1) $$l=k\sqrt{\frac{\sigma }{\mu }t}$$

(2) $$l_{m}(t)=k\sqrt{\left(1+\beta \frac{d}{\phi^{\frac{1}{3}}\omega }\frac{\cos\theta_{b}}{\cos\theta }\right)\frac{\sigma }{\mu }t}$$

where *k* is the proportional constant; *σ*, the surface tension; *μ*, the viscosity of the liquid; ω, the channel width; *ϕ*, the porosity of the paper; *d*, the pore diameter; *θ*, the contact angle; *θ_b_*, the contact angle on the side boundaries; *t*, the time; and *β* is introduced to consider the length of the advancing contact lines in contact with the hydrophobic side boundaries [87]. The modified Washburn equation has two coefficients (*k* and β) that should be determined experimentally by fitting a mathematical model on the experimental test results. Figure 5a represents paper channels with hydrophobic and laser-cut boundaries. Figure 5b illustrates our experimental results for hemp paper and fitted curves. Our experiment yielded R-squared values of 0.9535 and 0.9278 for laser-cut and hydrophobic boundaries on hemp paper (i.e., approximately 95.35% and 92.78% of the observed variation can be explained by the modified Washburn model for laser-cut and hydrophobic boundaries on hemp paper, respectively). Besides, the R-squared values for WG1 paper were calculated to be 0.9154 and 0.9470 for laser-cut and hydrophobic boundaries, respectively. Figure 5b demonstrates that the channels with hydrophobic side boundaries had shorter wicking distances on both papers, owing to the surface tension at the boundaries acting in an inverse direction to the flow direction. Moreover, we found that hemp paper-based channels had acceptable compliance with the modified Washburn equation, enabling the use of this equation in the design process for hemp-based microfluidic channels.

**Figure 6:**
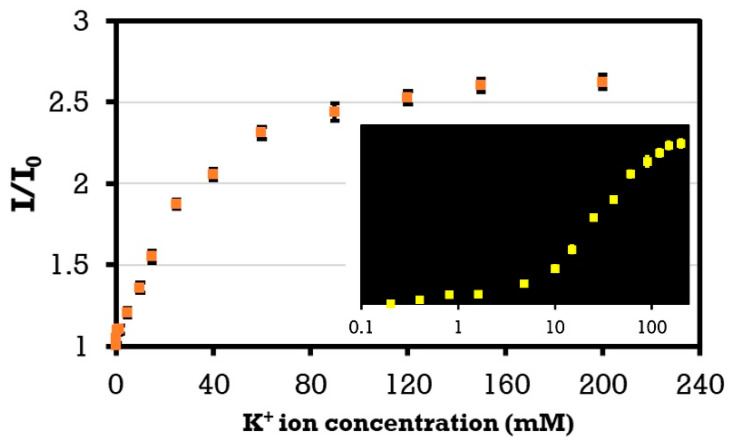
K+ cation measurement in artificial urine (pH 6) using PBFI potassium-sensitive dye as a fluorescent probe. Calibration curve for K+ ions on the hemp paper matrix at a constant probe concentration of 25 μM in DMSO (PBFI (λex/λem: 360/450 nm)). Error bars represent the standard error of the mean (n = 6).

Finally, we synthesized a solution of artificial urine with a wide range of ion concentrations following a protocol reported earlier by us and others [90]. We used a plate reader to quantify the fluorescent intensity of K+ cations. Figure 6 demonstrates the results of detecting K+ ions in the artificial urine. The fluorescent intensity ratio, as a function of the concentration, can be expressed as I/I_0_ = A-B*exp(−αC), where I and I_0_ are the intensities of the solution with and without the target ion, respectively; α is the saturation decay constant; C is the ion concentration in the solution; and A and B are constants. The results show that by increasing the concentration of potassium from 4.8 to 120 mM within the physiological range, the fluorescent probe intensity increased by 109.4%.

## 5. Discussion and Conclusions

Hemp is a sustainable, economical, renewable, and environmentally friendly substitute for the paper that has not been developed to its full potential due to the legal impediments, limited land under cultivation in the world, lack of infrastructures for processing the raw hemp to end products, and inadequate conducted studies exploring the drawbacks as well as the potential of hemp for prospective commercial products. This study characterized the capabilities of hemp as a substrate for the single-step, rapid prototyping of mass-producible paper-based microfluidic devices. Using a simple desktop pen plotter and markers, hydrophobic barriers (circles with 4 mm diameters) were plotted on hemp paper to examine the potential of the markers to form water-resistant patterns. Additionally, different plotting speeds and numbers of passes of the marker, as decisive factors for the amount of ink diffusing through the paper, were tested to find the optimized quantities for these factors. It was concluded that the Comix-broad tip and Deli-red markers can create water-resistant patterns at all speeds (1–110%) with three passes, except for 1%, which resulted in an ink-filled pattern. Conversely, Deli-black was not able to create water-resistant barriers at all, neither with changing the plotting speed nor with varying the number of passes. Furthermore, in order to achieve higher resolutions, the thinnest and smallest possible water-resistant patterns were investigated. The thinnest water-resistant pattern was achieved with the Comix-fine tip with a speed of 20% and two passes. The smallest plottable pattern, not being filled by ink, was a 1 mm-in-diameter circle, drawn with AxiDraw at the speed of 20% and two passes.

Moreover, the maximum wicking distance and time needed for wicking, as important parameters for determining the functionality of paper-based microfluidic devices, were compared for both hemp paper and WG1 paper. Upon examining different channel widths, no significant discrepancies between wicking distances were recorded for various channel widths with laser-cut boundaries on hemp paper. Therefore, thinner laser-cut channels can be used in hemp-based microfluidic devices to diminish the required amounts of pricey samples and reagents for tests. Nonetheless, channels with hydrophobic side boundaries experienced a decline in wicking distance as a result of narrowing down the channel width. In this regard, a modified version of the Washburn equation is presented, which was in consensus with our experimental results for the wicking length in hemp paper-based channels with hydrophobic boundaries. Hence, the modified Washburn equation can be used to design and predict the wicking length on hemp paper-based microfluidic devices. Besides, owing to the larger pore structure and higher absorbance capability, WG1 wicks viscous fluids faster than hemp paper. However, their performance was comparable for low-viscosity fluids such as urine. Hence, in order to overcome the lower wicking speeds in hemp, we suggest increasing the packing density of hemp-based microfluidic devices by simple techniques such as origami, adhesives, or high-resolution patterning [80]. Densely packed devices benefit from shorter channels from the input to reaction zones, decreasing the time required for testing with hemp-based devices. Furthermore, the wettability of hemp and WG1 paper was compared by measuring their contact angles, proving a similar capability of hemp to WG1 paper regarding the hydrophobic nature of the paper. In addition, the effect of branching on the wicking distance was studied on hemp paper, showing no significant discrepancy between the wicking distances of a single and four-branch channel for the first 60 s of wicking. However, for the same channels, after 180 s, the wicking distance of a single channel was virtually 18% more than the four-branch channel.

The modifiable pore size, biocompatibility, and porous structure of paper provide a natural support medium, emulating the cellular microenvironment physically, for culturing cells for disease model construction, cell differentiation, and single-cell analysis [92]. However, although the use of different materials (e.g., Teflon [93]) was reported to enhance cell adhesion, the adhesion of cells to the paper substrate is still a challenge to be addressed, particularly in long-term cultures (more than a week) [92]. Furthermore, the paper substrate has limitations for cell applications that demand continuous perfusion [92]. Hybrid microfluidic devices have been reported [94,95] to overcome this limitation, while further research may extend the applicability of paper-based microfluidic devices for cell culture and tissue engineering applications. The integration of microfluidics with electronic sensors can offer higher sensitivity [96]. The inkjet printing of silver nanoparticles, to produce high-resolution conductive electronic sensors on nitrocellulose, was reported previously [97]. The main challenges were achieving lower printing resolutions and uninterrupted conductivity [98]. Future studies could evaluate the possibility of printing conductive material on hemp substrates to form electronic sensors. Overall, intriguing topics for future research could include exploring other possible applications of hemp in the fabrication of low-cost test devices, employing machine-learning methods [99] to quantify hemp-based assays more accurately, solving present issues (e.g., slow wicking speeds), and developing startups to fabricate innovative products such as hemp-based menstrual pads [100] that are UV-resistant, anti-bacterial/inflammatory/fungal, reusable, and biodegradable.

## Supplementary Materials

The following are available online at https://www.mdpi.com/2072-666X/12/2/182/s1, Figure S1: images of patterned hemp paper with different markers, Figure S2: images of wicking of fluid on hemp paper channels, Figure S3: images of fluid wicking on branched hemp paper, Figure S4: images of wicking of solutions with different viscosities on hemp paper.
