# Supplementary material for: Hemp-Based Microfluidics
Source: Micromachines (Basel). 2021 Feb 12;12(2):182. doi: 10.3390/mi12020182 (PMC7917756; doi:10.3390/mi12020182)
Supplement: Supplementary file 1 [file micromachines-12-00182-s001.pdf]

Supplementary File

Hemp-based microfluidics

Mikail Temirel, Sajjad Rahmani Dabbagh and S. Tasoglu

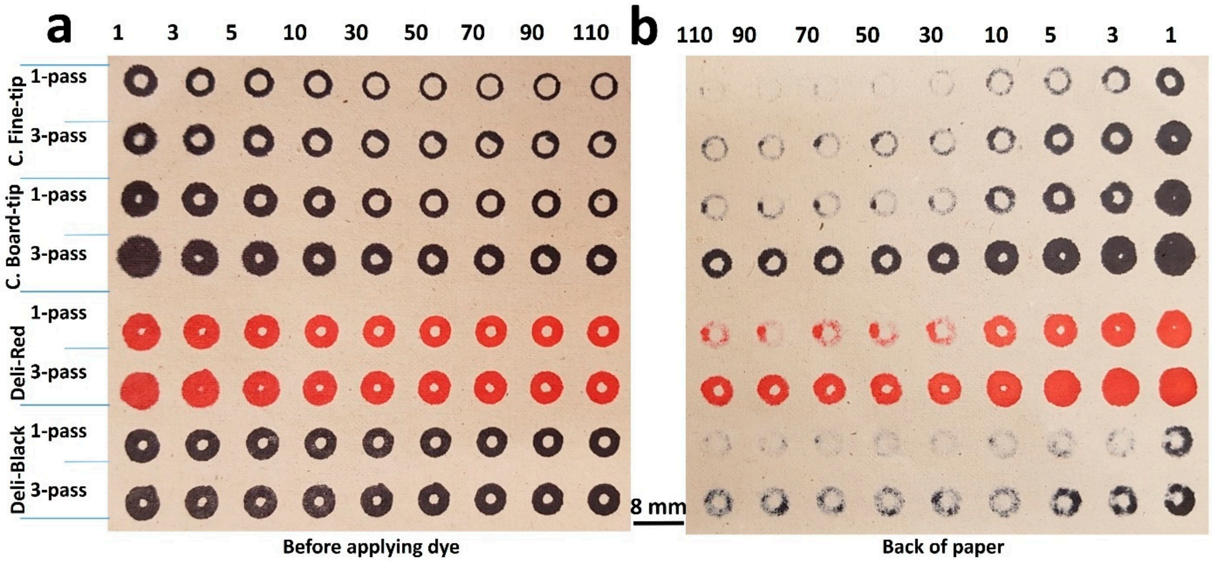

Figure S1. Images of 4 mm in diameter patterns plotted by AxiDraw using several markers including Comix (fine and board tips) and Deli (red and black colors) brand markers. Different plotting speeds, ranging from 1% to 110%, and multiple passes were tested. **(a)** The front view of plotted patterns before applying aqua dye. **(b)** Back view of the plotted patterns, displaying the amount of penetrated ink through the thickness of hemp paper.

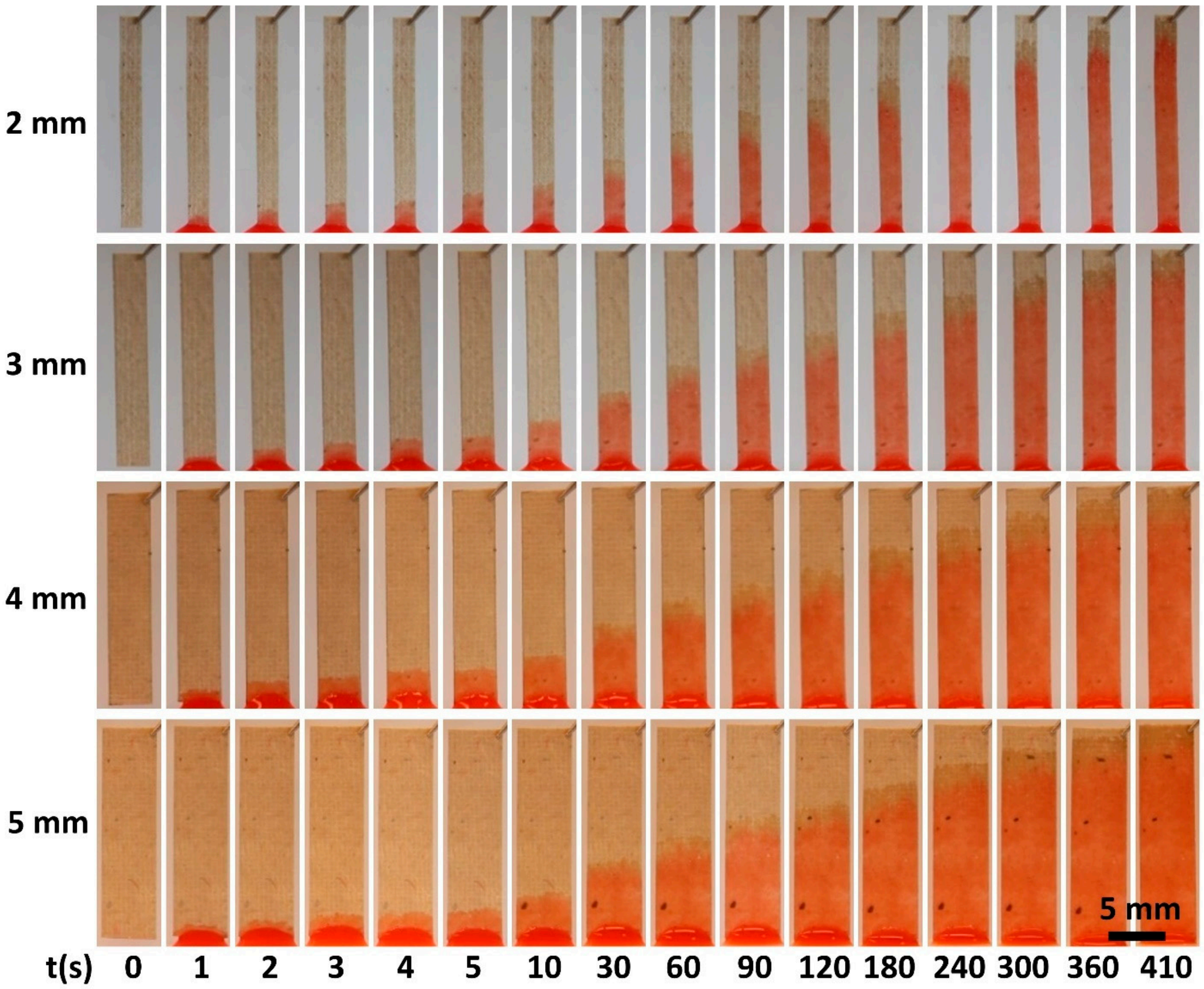

Figure S2. Representative images of wicking of fluid on hemp paper channels with varying channel widths from 2 mm to 5 mm over 410 s.

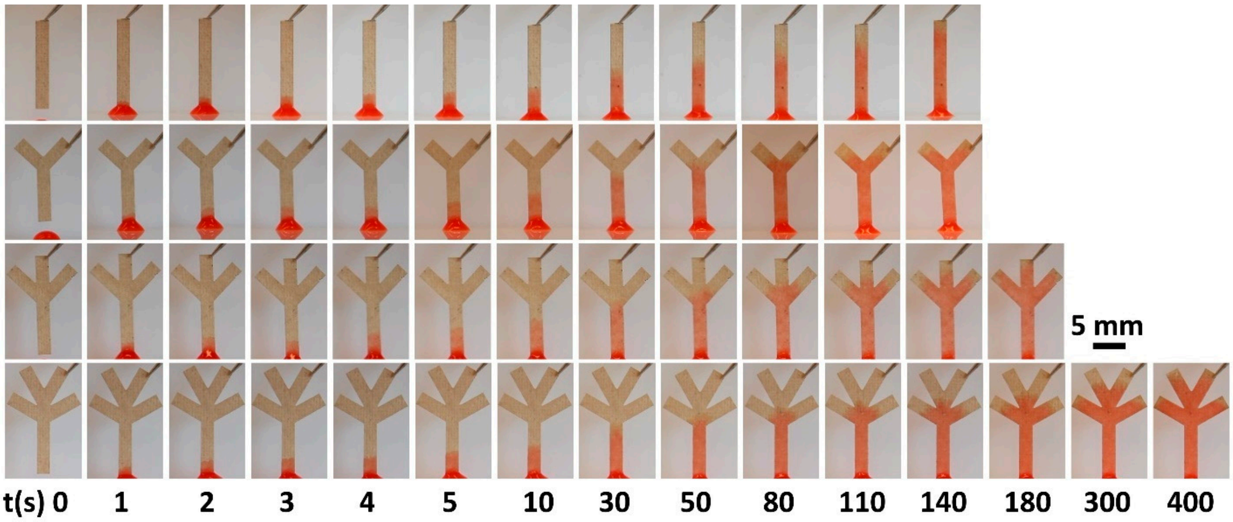

Figure S3. Representative images of fluid wicking on hemp paper with different number of branches, from 1 to 4, over 400 s through 2 mm width channels .

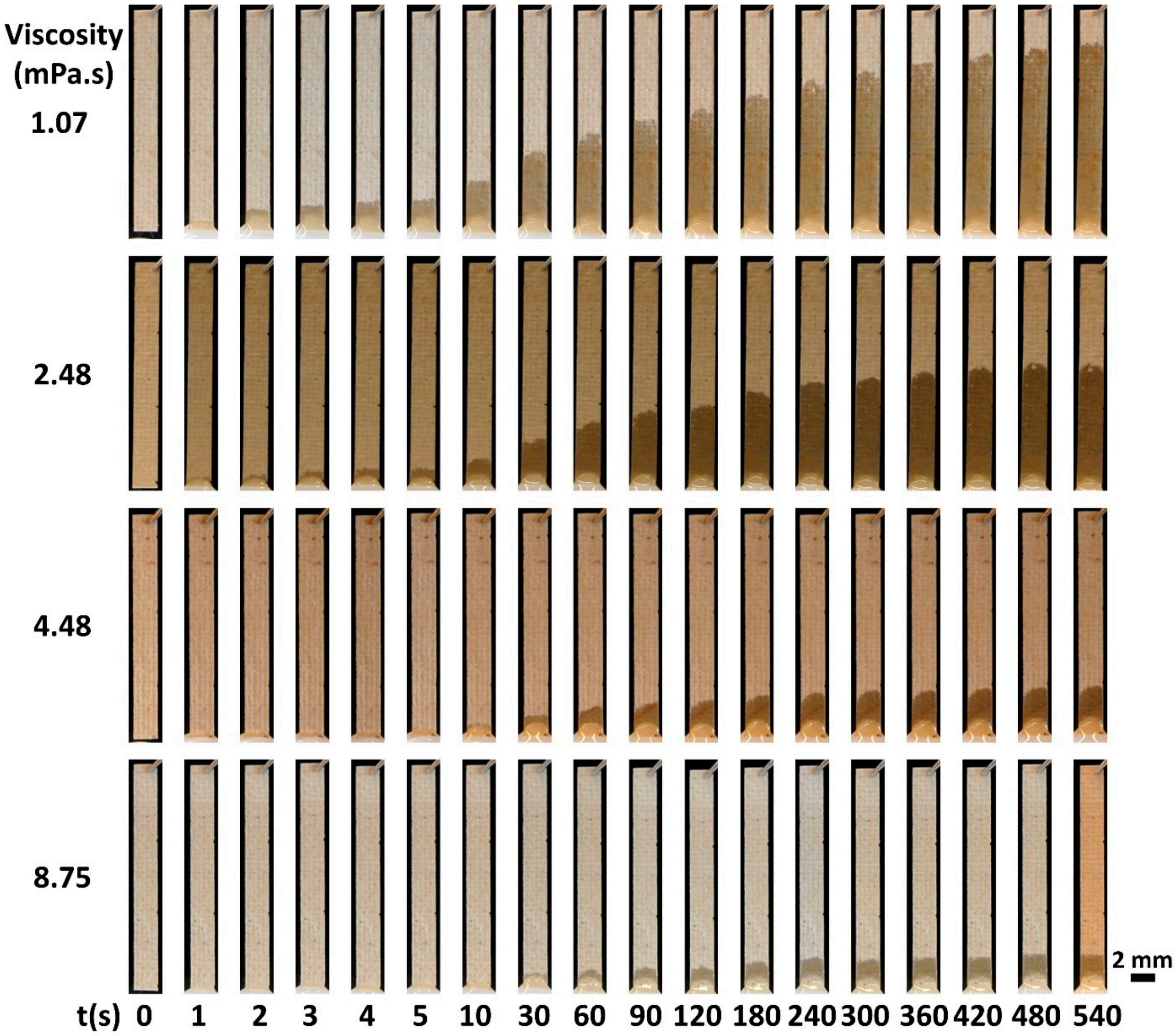

Figure S4. Representative images of wicking of solutions with different viscosities, from 1.07 to 8.75 mPa.s, on hemp-based microfluidic channel with a constant channel width of 2 mm .

© 2021 by the authors. Submitted for possible open access publication under the terms and conditions of the Creative Commons Attribution (CC BY) license (http://creativecommons.org/licenses/by/4.0/).
